# Effects of 15 weeks of resistance exercise on pro-inflammatory cytokine levels in the vastus lateralis muscle of patients with fibromyalgia

**DOI:** 10.1186/s13075-016-1041-y

**Published:** 2016-06-13

**Authors:** Malin Ernberg, Nikolaos Christidis, Bijar Ghafouri, Indre Bileviciute-Ljungar, Monika Löfgren, Anette Larsson, Annie Palstam, Jan Bjersing, Kaisa Mannerkorpi, Eva Kosek, Björn Gerdle

**Affiliations:** Department of Dental Medicine, Karolinska Institutet, and Scandinavian Center for Orofacial Neurosciences (SCON), SE-141 04 Huddinge, Sweden; Pain and Rehabilitation Centre, and Department of Medical and Health Sciences, Linköping University, SE-581 85 Linköping, Sweden; Department of Clinical Sciences, Karolinska Institutet, and Department of Rehabilitation Medicine, Danderyd Hospital, SE-182 88 Stockholm, Sweden; Institute of Neuroscience and Physiology, Section of Health and Rehabilitation, Physiotherapy, Sahlgrenska Academy, University of Gothenburg, SE-405 30 Göteborg, Sweden; University of Gothenburg Centre for Person-centred Care (GPCC), Sahlgrenska Academy, SE-405 30 Göteborg, Sweden; Department of Rheumatology and Inflammation Research, Institute of Medicine, Sahlgrenska Academy, University of Gothenburg, SE-405 30 Göteborg, Sweden; Department of Clinical Neuroscience, Karolinska Institutet, SE-171 77 Stockholm, Sweden; Stockholm Spine Center, SE-194 89 Stockholm, Sweden

**Keywords:** Cytokines, Dynamic contractions, Exercise, Fibromyalgia, Microdialysis, Quadriceps

## Abstract

**Background:**

This study aimed at investigating the effect of a resistance exercise intervention on the interstitial muscle levels of pro-inflammatory cytokines in fibromyalgia (FMS) and healthy controls (CON).

**Methods:**

Twenty-four female patients with FMS (54 ± 8 years) and 27 female CON (54 ± 9 years) were subjected to intramuscular microdialysis of the most painful vastus lateralis muscle before and after 15 weeks of progressive resistance exercise twice per week. Baseline dialysates were sampled in the resting muscle 140 min after insertion of the microdialysis catheter. The participants then performed repetitive dynamic contractions (knee extension) for 20 min, followed by 60 min rest. Pain intensity was assessed with a 0–100 mm visual analogue scale (VAS), and fatigue was assessed with Borg’s RPE throughout microdialysis. Dialysates were sampled every 20 min and analyzed with Luminex for interleukin (IL)-1β, tumor necrosis factor (TNF) alpha, IL-6, and IL-8.

**Results:**

At both sessions and for both groups the dynamic contractions increased pain (*P* < 0.012) and fatigue (*P* < 0.001). The levels of TNF were lower in the FMS group than the CON group at both sessions (*P* < 0.05), but none of the other cytokines differed between the groups. IL-6 and IL-8 increased after the dynamic contractions in both groups (*P* < 0.010), while TNF increased only in CON (*P* < 0.05) and IL-1β did not change. Overall pain intensity was reduced after the 15 weeks of resistance exercise in FMS (*P* < 0.05), but there was no changes in fatigue or cytokine levels.

**Conclusion:**

Progressive resistance exercise for 15 weeks did not affect the interstitial levels of IL-1β, TNF, IL-6, and IL-8 in the vastus lateralis muscle of FMS patients or CON.

**Trial registration:**

Clinicaltrials.gov NCT01226784, registered 21 October 2010.

## Background

Chronic pain is a huge health problem worldwide with immense costs for the society in terms of lost productivity, health care visits, medication, and so forth, but also for the individual in terms of social burden and negative impact on quality of life [1]. In the USA, as many as 100 million adults suffer from chronic pain with an estimated cost of around US$600 billion [1, 2]. In Europe, almost 20 % of the population report pain of moderate to severe intensity with at least a 6-month duration, with an estimated yearly cost of €3.4 billion [3]. Musculoskeletal pain is by far the most frequent type of chronic pain, encompassing about 90 % of all chronic pain [3, 4].

Fibromyalgia syndrome (FMS) is a musculoskeletal pain disorder that mainly affects women and is characterized by widespread pain and tenderness, stiffness, fatigue, sleep disturbances, and cognitive dysfunction [5]. Exercise intolerance is regarded a key symptom in FMS [6] and even normal household chores often lead to worsening of symptoms. In healthy subjects, exercise usually increases pressure pain thresholds (PPTs) due to activation of endogenous analgesia, but in FMS patients muscle contractions reduce PPTs [6–8]. In spite of this, physical exercises of moderate intensity are included in the multimodal intervention programs for the management of FMS. Indeed, several studies have shown beneficial effects of regular exercises in FMS [9, 10]. Further, exercise of moderate intensity increased functional aerobic capacity and improved activities of daily living in FMS [11] and a recent systematic review reported positive results of diverse exercise interventions on pain, multidimensional function, and self-reported physical function [12].

Despite its large impact on society, the mechanisms behind FMS are not well known. A biopsychosocial model is mostly suggested, including both biological and psychological factors. With respect to biological factors, the common belief has for some time been that central alterations in pain processing are of greatest importance and that peripheral input plays a minor or no role [13]. However, it was recently suggested that peripheral mechanisms, such as temporal summation, due to normal muscle work may be important to drive pain hypersensibility [14–16]. This may be enough to cause repeated release of nociceptive and inflammatory substances causing long-lasting peripheral and central sensitization. In support of this is increasing evidence over the past years that the interstitial levels of many algesic and metabolic substances are elevated in painful muscles of patients with local or regional chronic myalgia [17]. However, relatively few studies have investigated interstitial muscle levels of nociceptive substances in FMS. An early study showed that the release of serotonin in the masseter muscle was elevated in response to trauma in FMS [18]. More recently, Gerdle et al. showed that the interstitial levels of lactate and pyruvate in the trapezius muscle were elevated in patients with FMS [19, 20]. Patients with chronic widespread pain also seem to have difficulties in recruiting antinociceptive substances, such as endocannabinoids, in response to contractions [21]. An increased release of nociceptive substances in combination with reduced release of antinociceptive substances may therefore participate in pain amplification.

Cytokines are small proteins that are produced in many diverse cells of the immune system, such as monocytes, lymphocytes, and mast cells, but also in endothelial cells and fibroblasts [22]. They are involved in cell signaling and play a critical role in the immune system. Many of the symptoms seen in inflammatory conditions that are generally attributed to central release of cytokines are similar to those seen in FMS [22]. For example, animal research suggests a role for interleukin (IL)-1β in fatigue and hyperalgesia, and tumor necrosis factor (TNF) beta and IL-6 can trigger daytime sleepiness and pain but also elicit cognitive dysfunction [22–25]. This may imply that central release of cytokines may be involved in FMS. Indeed, central inflammation was proposed in its pathophysiology based on findings of increased cerebrospinal fluid (CSF) levels of IL-8 in FMS [26].

It is well-known that short-term exercise increases some cytokine levels, especially IL-6 [27]. Some studies have investigated circulating cytokine levels in FMS and reported increased levels of IL-6, IL-8, and TNF, but a blunted response of IL-10 to short-term exercises [6, 28]. We have recently investigated the effect of dynamic muscle contractions on interstitial muscle release of pro-inflammatory cytokines in FMS [29]. We found that TNF was higher in pain-free controls than in FMS and increased in response to contractions only in controls, while IL-6 and IL-8 did not differ between groups and increased in both groups. However, muscle and plasma cytokine levels were not related.

Interestingly, while short-term exercise seems to increase cytokine levels, a few recent reports have shown reductions in pro-inflammatory cytokine levels in response to long-term exercise in FMS. A systematic review based on nine articles concluded that, despite minimal evidence, exercise interventions might act as an anti-inflammatory treatment in FMS patients by reducing circulating IL-6 and IL-8 levels [30]. Further, in FMS patients subjected to 15 weeks of Nordic walking, changes of insulin growth factor-1 (IGF-1) correlated positively to changes of pain threshold, indicating a possible beneficial role for IGF-1 during exercise [31].

However, no previous study has investigated long-term effects on muscle levels of cytokines in response to physical exercise in FMS. Therefore, this study aimed at investigating the interstitial muscle release of pro-inflammatory cytokines in patients with FMS before and after a resistance exercise intervention. The hypothesis was that if muscle release of pro-inflammatory cytokines was elevated in FMS, they would be normalized after 15 weeks of resistance exercise.

## Methods

### Participants

Female patients with FMS and age-matched healthy women (CON) were included. The subjects participated in a randomized controlled multicenter trial regarding the effects of progressive resistance exercise or relaxation exercise on FMS symptoms (Clinicaltrials.gov NCTO1226784, 21 October 2010). The enrollment process for the patients has been described in detail previously [32, 33].

Microdialysis was performed in a subgroup of patients and CON. The first 10 patients at each of the three participating centers (12 at one site) who were randomized to resistance exercise and volunteered to also participate in microdialysis before and after the exercise were included. For CON, healthy individuals in the multicenter center study who were willing to participate in microdialysis and resistance exercise were included (the same number as FMS). They were matched to the FMS patients according to age. In this study, data are reported for those subjects who participated in both microdialysis sessions and for whom complete microdialysis data were obtained at both sessions. Two FMS patients and two CON of the included 64 participants dropped out from the study and additionally six FMS patients and three CON were excluded due to technical failure during microdialysis (e.g., crashed catheters, so no fluid could be sampled). Thus, 24 FMS patients and 27 CON participated.

Inclusion criteria for the patients were: 1) a diagnosis of FMS according to the American College of Rheumatology (ACR) 1990 [34] classification criteria; and 2) to be of working age (20–65 years).

Inclusion criteria for the healthy controls were: 1) good general health; 2) pain-free; and 3) age 20–65 years.

Exclusion criteria for both groups were: 1) high blood pressure (>160/90 mmHg); 2) osteoarthritis in the hip or knee; 3) other severe somatic or psychiatric disorders; 4) primary causes of pain other than FMS; 5) high consumption of alcohol (audit >6); 6) participation in a rehabilitation program within the past year; 7) regular resistant exercise training or relaxation exercise training more than twice a week; 8) inability to understand or speak Swedish; and 9) not being able to refrain from non-steroidal anti-inflammatory drugs (NSAIDs) for 1 week and paracetamol, opioids, or hypnotics for 48 h prior to microdialysis.

The participants were allowed to continue with their other regular medication. As such, eight in the FMS group used antidepressants (tricyclic antidepressants (TCA), selective serotonin reuptake inhibitors (SSRI), serotonin norepinephrine reuptake inhibitors (SNRI)), one used gabapentin, and one used tramadol. Three CON used antidepressants (SSRI).

### Procedure

The participants were examined clinically by a physician approximately 1 week before the first microdialysis session. The examination also included a functional test of physical capacity, the number of tender points, pressure algometry, and a battery of questionnaires. The questionnaires included registrations of psychological distress and quality of life. For all participants age (years), weight (kg), height (m) and blood pressures (mmHg) were registered and body mass index (BMI; kg/m^2^) was calculated.

Intramuscular microdialysis in the vastus lateralis muscle of the most painful leg was performed during 220 min before and after the resistance exercise. Before microdialysis, a venous blood sample (20 mL) was drawn from the decubital vein for analysis of plasma cytokine levels for comparison to interstitial levels, i.e., to see if interstitial levels indicate local release. Both sessions included 20 min of repetitive dynamic muscle contractions of the leg during microdialysis.

### Assessment of anxiety, depression, and quality of life

All participants completed a battery of questionnaires; of these the validated Swedish versions of the Hospital Anxiety and Depression Scale (HADS) to assess anxiety and depression [35], and the Short-Form Health Survey (SF36) to assess quality of life [36] were included as background variables in this study. For a complete description of the HADS and SF36, see [37, 38]. From the SF36 the physical (SF36-PSC) and mental (SF36-MSC) summary components were calculated.

### Physical capacity

Three tests were used for measurement of physical capacity: maximal isometric elbow flexion force, maximal isometric knee extension, and a 6-min walk test (6MWT). Maximal isometric elbow flexion force (kg) was measured in both arms (one at a time) using a dynamometer (Isobex®; Medical Device Solutions AG, Oberburg, Switzerland) with the participant seated without back support with legs stretched out in front. The upper arm was aligned with the trunk and the elbow was bent in 90° flexion*.* The maximum force obtained during a period of 5 s was recorded [39].

Static knee extension force (N) was determined in both legs using a dynamometer (Steve Strong®; Stig Starke HBI, Göteborg, Sweden). The participants were seated in a fixed position with back support and the knee and hip in 90° of flexion and legs hanging freely. A non-elastic strap was placed around the ankle and attached to a pressure transducer with an amplifier. The maximum force during a period of 5 s was recorded. The instrument has been used in previous studies of physical performance [40, 41].

For the 6MWT, the distance that the participant could walk during six min in a standardized situation was registered [42].

### Pressure algometry

Pressure pain thresholds (PPTs), defined as the minimum pressure that elicited a pain response, were recorded bilaterally over four points in FMS in accordance with the ACR 1990 criteria [34], i.e., the supraspinatus muscle (at origins above the scapula spine near the medial border), the lateral epicondyle (2 cm distal to the epicondyles), the gluteus maximus (in the upper outer quadrants of the buttocks in the anterior fold of muscle), and the inside of the knee (at the medial fat pad proximal to the joint line). Each site was assessed only once to avoid the risk of temporal summation and the same order (as described above) was used for all participants starting with the points on the right side. An electronic algometer (Somedic Sales AB, Höör, Sweden) with a blunt rubber tip of 1 cm^2^ and a pressure rate of approximately 50 kPa/s was used. Before the recordings, the subjects were given instructions and the procedure was tested over a site not included in the actual recordings. The algometer was held perpendicular to the skin at the site and subjects were instructed to press the button when the sensation of pressure changed to pain [43]. If the subject did not press the button before 1500 kPa was reached, the application of pressure was interrupted. The mean value of all eight assessments for each subject was calculated and used for further analysis as the individual’s PPT.

### Assessments of pain and fatigue

Current pain intensity was assessed as overall pain and pain for the most painful leg separately with a 0–100 mm visual analogue scale (VAS), with the endpoints 0 = no pain and 100 = worst imaginable pain. The level of fatigue was assessed with Borg’s 6-20 Ratings of Perceived Exertion (Borg’s RPE) Scale [44].

### Microdialysis

The participants were instructed to refrain from nicotine (smoking) and caffeine (coffee or tea), but otherwise to eat breakfast as usual in the morning on the day of microdialysis. When they arrived at the clinical department it was ensured that they had not taken any NSAIDs during the last 7 days and no analgesics or sleeping pills in the last 48 h.

During microdialysis, the participants were resting in a supine position on a bed, except during the 20 min of dynamic contractions, when they were sitting on the bed with the back supported. The participants were allowed to drink water and were served a cheese sandwich after 1.5 h (during trauma phase, before the dynamic contractions).

Microdialysis was performed in the vastus lateralis muscle of the most painful leg. If both legs had similar pain levels, the muscle on the dominant side was chosen. The same muscle was used at both sessions. The skin overlying the muscle was anesthetized with 0.5 mL lidocaine (Xyolocain 20 mg/mL). Two commercially available microdialysis catheters (CMA Microdialysis AB, Solna, Sweden), with 20 kDa (CMA 60) and 100 kDa (CMA 71) cut-off and membrane length 30 mm, were inserted into the muscle 3 cm apart in parallel to the muscle fibers in the mid-third part of the distance from the trochanter to the knee joint 5 min after skin surface anesthesia. The 100 kDa catheter was used for sampling cytokines in the present study and the 20 kDa for metabolites (data presented elsewhere). The catheters were perfused with an infusion pump (CMA 107) set at 5 μL/h with a Ringer acetate solution (Fresenius Kabi AB, Uppsala, Sweden) containing 3 mM glucose, 0.5 mM lactate, 3.0 μM [^14^C]-lactate (specific activity: 5.81 GBq/mmol; GE Healthcare, Buckinghamshire, UK) as well as 0.3 μl/ml ^3^H_2_O (specific activity: 37 MBq/g; PerkinElmer Life Sciences, Boston, USA).

Microdialysates were sampled every 20 min. The first 120 min of microdialysis was regarded as the trauma phase and dialysates from this period were not analyzed. The dialysate sample obtained at 140 min was regarded as the baseline. The subjects then performed repetitive dynamic contractions of the quadriceps muscle (knee extension) for 20 min. The participants were seated with the legs outside the bed with the exercising leg slightly bent and the foot and calf placed on a 40 cm Pilatus ball. For participants with baseline pain level ≥40 mm on the VAS in the exercising leg a knee angle of 15° was used and for those with pain levels <40 mm on the VAS an angle of 20° was used. The contralateral leg was bent 90° with the foot supported. During the exercise the participants performed continuous knee extensions by slowly lifting the leg until straight and then lowering it down on the Pilatus ball, and then immediately lifting it again without resting the leg on the ball. Each such cycle of dynamic contractions took 5 s and was repeated for 20 min. After the contractions the subjects were asked to lie down again on the bed and rest while the microdialysis continued for 60 min.

Pain intensity (VAS) was assessed before insertion of the microdialysis catheter and every 20 min (when changing microdialysate vials) throughout the microdialysis session, but every 5 min during the 20-min dynamic contractions. Fatigue (Borg’s RPE) was assessed at baseline (140 min) and every 5 min during the dynamic contractions.

### Resistance exercise intervention

Previous studies have shown that low-intensity exercise, adjusted to individual limitations, is feasible for most FMS patients [45]. Progressive resistance exercise, twice per week for 15 weeks under the supervision of specially trained physical therapists, was used in this study. Each session started with 10 min bicycling to warm up and was then followed by 50 min resistance exercise. The exercise was initiated at low loads at 40 % of the maximum voluntary capacity (MVC) and successively progressed up to 70–80 % of MVC [46].

### Handling of blood and microdialysate samples and biochemical analyses

The blood samples were placed on ice and immediately transported to the laboratory where they were centrifuged (1500 g) for 30 min. The plasma was pipetted into 1.5 mL Eppendorf vials and frozen (–70 ° C) until analyses. All microdialysate vials were weighed before and after microdialysis to confirm that fluid recovery was adequate. After visual inspection for blood contamination and weighing, the samples were removed to Eppendorf tubes and immediately frozen (–70 ° C).

Dialysate and plasma samples were analyzed for IL-1β, TNF, IL-6, and IL-8 with Luminex technology (Bioplex, Biorad Laboratories Inc., Hercules, CA, USA) using standard kits with magnetic beads (dialysate: Millipore HSCYTMAG-60SK; and plasma: HSTCMAG-28SK; Merck KGaA, Darmstadt, Germany). The limits of detection were: IL-1β <0.14 pg/mL; IL-6 < 0.13 pg/mL; IL-8 < 0.13 pg/mL; and TNF <0.16 pg/mL. The intra-assay percent coefficient of variation (%CV) was <10 % and the inter-assay %CV <20 % for the analytes in both kits.

### Statistics

Statistical analyses were made using Statistica version 12 (StatSoft Inc., Tulsa, OK, USA) and SigmaPlot version 13 (SysStat Inc., San José, CA, USA). The Kolmogorov-Smirnov test was used to test for normality of the data. None of the cytokines were normally distributed. An attempt was therefore made to logarithmic (ln) transform data, but data remained non-normally distributed. Most other data were not measured on a continuous scale, and non-parametric statistics were used for all analyses. To analyze differences between cytokine levels, pain intensities, and fatigue between sessions (before and after the exercise intervention), the average of the cytokine levels (cytokine_AV_) as well as pain (pain_AV_) and fatigue (fatigue_AV_) assessments obtained throughout the microdialysis were used.

Descriptive data are presented as mean and standard deviation (SD) or median and interquartile range (IQR). Data before and after exercise were compared in each group separately with Wilcoxon’s test or Friedman’s ANOVA, whereas between-group comparisons were made with independent *t* test or Mann Whitney *U* test. Dunn’s test for multiple comparisons against a control group (baseline) was used as a post-hoc test when the Friedman test was significant. The Spearman correlation test was used to analyze associations between cytokine levels and pain as well as fatigue during microdialysis. A significance level of *P* < 0.05 was used in all analyses.

## Results

### Dialysate cytokine levels

**Fig. 1:**
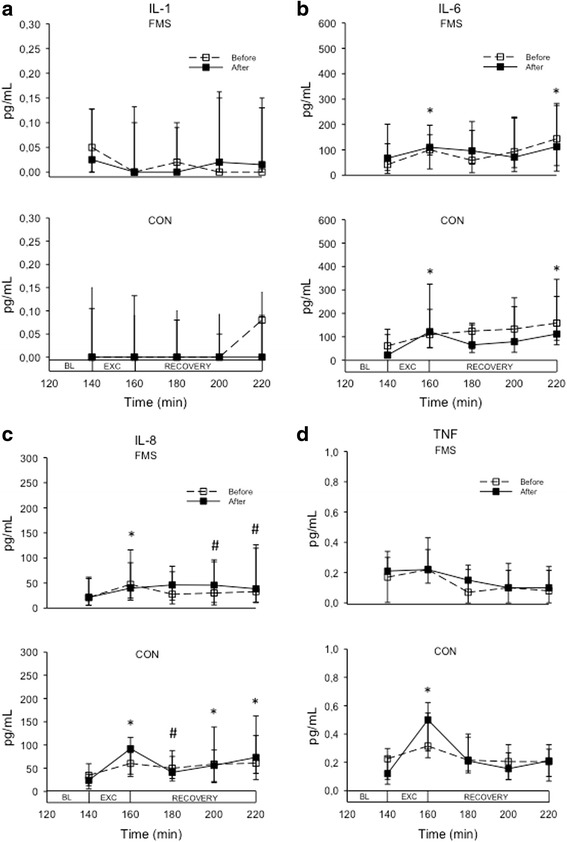
Line and scatter plot showing the median (interquartile range) levels of cytokines during microdialysis in the vastus lateralis muscle of the most painful leg in 24 patients with fibromyalgia (*FMS*) and 27 healthy controls (*CON*) before and after 15 weeks of resistance exercise twice per week. **a** IL-1β, **b** IL-6, **c** IL-8, **d** TNF. Microdialysis was performed for 220 min. During the first 140 min the participant rested (data not shown), whereafter they performed dynamic contractions of the leg (knee extension) for 20 min (*EXC*) followed by another 60 min of rest (recovery phase). Samples were collected every 20 min and the dialysate sampled between 120–140 min was regarded as baseline (*BL*). *P < 0.05, both sessions; ^#^ *P* < 0.05, only after resistance exercise. *IL* interleukin, *TNF* tumor necrosis factor beta

The cytokines levels are shown in Table 1 and Fig. 1. All cytokines were detectable in the dialysates, but in varying frequencies with IL-1β being detectable in less than 50 % of samples, TNF in approximately 80 %, and IL-6 as well as IL-8 in more than 90 %. There were more samples with detectable levels of TNF in CON than FMS, but otherwise no group differences (Table 1). The cytokine levels varied considerably between participants, and IL-1β and TNF in general showed very low levels. The cytokine levels further showed different profiles in response to the dynamic contractions.

**Table 1:** Dialysate and plasma levels (pg/mL) of cytokines as well as frequency (%) of detectable samples in 24 patients with fibromyalgia (FMS) and 27 healthy controls (CON) before and after 15 weeks of resistance exercise twice per week, as well as the *P* value for within-group comparison (Wilcoxon test)

		FMS		*P* value	CON		*P* value
		Before	After		Before	After	
IL-1β							
Dialysate							
	Detectable	48	44		41	34	
	Level	0.10 (0.13)	0.05 (0.16)	0.522	0.06 (0.17)	0.01 (0.16)	0.479
Plasma							
	Detectable	70	83		89	96	
	Level	1.13 (3.55)	1.23 (1.86)	0.823	1.23 (1.40)	1.54 (1.33)	0.845
IL-6							
Dialysate							
	Detectable	97	95		98	100	
	Level	81.1 (131.7)	100.4 (236.3)	0.540	156.4 (196.9)	107.0 (173.5)	1.000
Plasma							
	Detectable	91	100		96	96	
	Level	1.60 (1.48)	2.35 (2.14)*	0.831	1.31 (1.43)	1.41 (1.24)	0.556
IL-8							
Dialysate							
	Detectable	95	91		98	99	
	Level	37.5 (75.6)	42.2 (71.6)	0.838	61.0 (101.4)	61.7 (58.0)	1.000
Plasma							
	Detectable	91	87		74	78	
	Level	1.66 (2.10)*	1.22 (3.19)	0.136	0.47 (1.61)	0.56 (1.11)	1.000
TNF							
Dialysate							
	Detectable	67^#^	67^#^		93	94	
	Level	0.16 (0.18)*	0.19 (0.25)*	0.831	0.27 (0.23)	0.26 (0.17)	0.556
Plasma							
	Detectable	100	100		100	100	
	Level	4.27 (1.70)	3.79 (3.67)	0.831	3.72 (2.68)	3.56 (2.31)	1.000

The dialysate levels represent the average of 5 samples obtained during microdialysis (140-220 min), which included 20 min of dynamic exercise. Dialysate and plasma levels are presented as median (interquartile range)

Between-group comparisons: *P < 0.05 (Mann-Whitney *U* test) ^#^ *P* < 0.001 (Chi square test)

*IL* interleukin, *TNF* tumor necrosis factor beta

The levels of IL-1β did not change during dynamic contractions in any group at any session.

IL-6 showed a similar profile in FMS and CON and changed significantly during both sessions (Friedman test: FMS, *P* < 0.008; CON, *P* < 0.001). In both sessions and both groups IL-6 was significantly increased in the sample obtained directly after the dynamic contractions compared to baseline, then dropped in the next sample whereafter it increased again and was significantly higher compared to baseline in the last sample (220 min).

IL-8 showed a similar profile to IL-6. It increased during dynamic contractions in both groups at both sessions (Friedman test: *P* < 0.008 both groups), but with a much less pronounced increase in FMS than CON, especially at the second session, i.e., after resistance exercise. The post-hoc test showed that it was significantly increased at most time points after the contractions compared to baseline in both groups (Fig. 1).

TNF showed a somewhat different profile in FMS than CON. Although it changed significantly in response to dynamic contractions at both sessions in both groups (Friedman test: FMS, *P* < 0.033; CON, *P* < 0.001), it was significantly increased after dynamic contractions only in FMS (Fig. 1).

When the cytokine levels were compared between sessions, TNF_AV_ was significantly lower in FMS than CON at both sessions, but otherwise no group differences were found in cytokine levels. None of the cytokines differed between sessions (Table 1).

### Plasma cytokine levels

All cytokines were detectable in plasma in more than 70 % of samples and without group differences in frequencies (Table 1). As with dialysate levels, there was a large variation in plasma levels between participants.

The plasma level of IL-8 at baseline (before exercise) was higher in FMS than CON, while plasma IL-6 was higher in FMS than CON after exercise. The plasma levels of IL-6 and IL-8 were lower than dialysate levels, while plasma IL-1β and TNF levels were higher than dialysate levels. Plasma levels did not change after resistance exercise in any of the groups (Table 1).

There were no significant correlations between plasma and dialysate levels at any session in any group.

### Pain intensity and fatigue

**Fig. 2:**
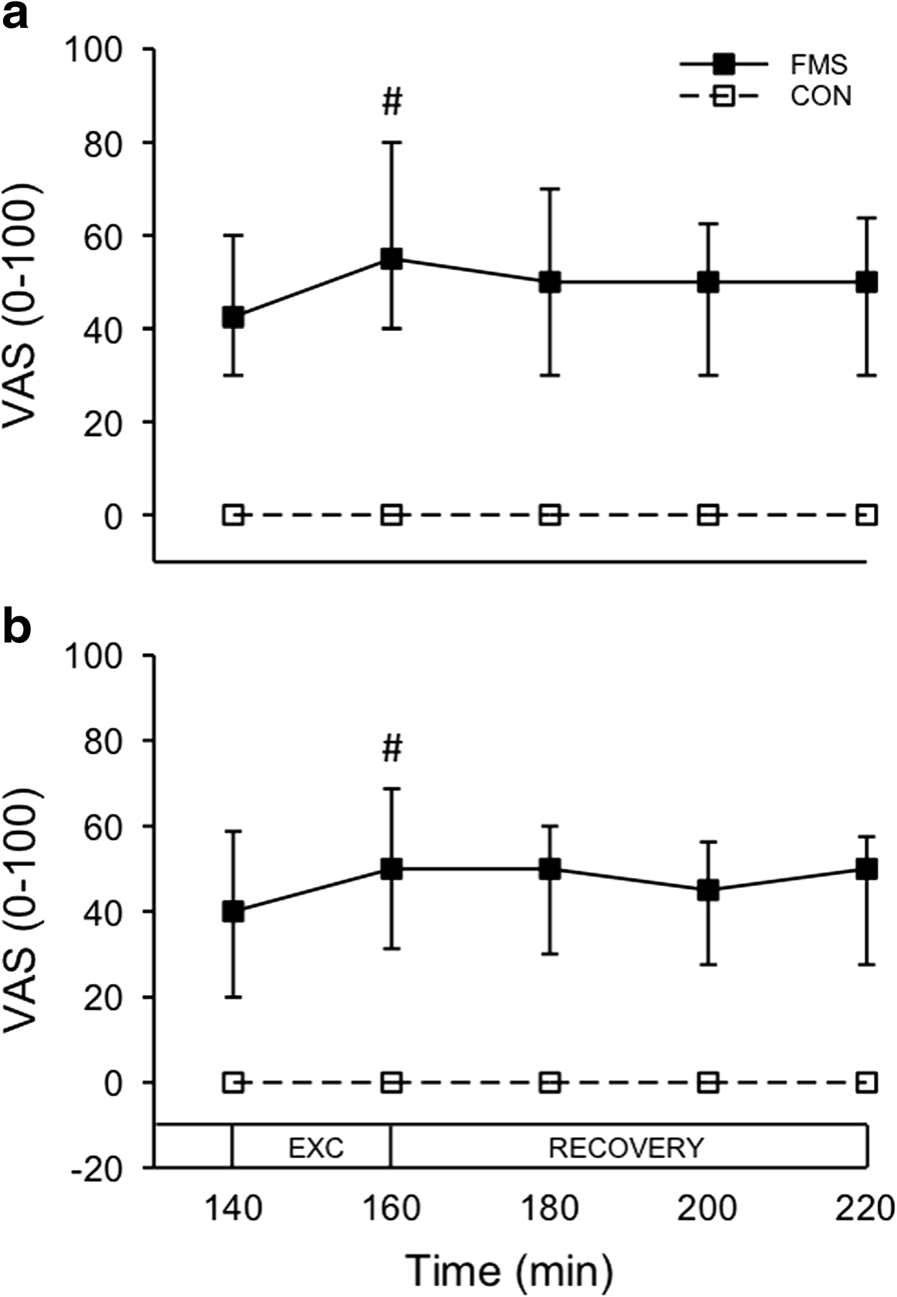
Line and scatter plot showing the median (interquartile range) pain intensity (VAS 0–100 mm) during microdialysis in the vastus lateralis muscle of the most painful leg in 24 patients with fibromyalgia (*FMS*) and 27 healthy controls (*CON*) before (**a**) and after (**b**) 15 weeks of resistance exercise twice per week. Microdialysis was performed for 220 min. During the first 140 min the participant rested (data not shown), whereafter they performed dynamic contractions (*EXC*) of the leg (knee extension) for 20 min followed by another 60 min of rest (*RECOVERY*). In FMS, but not in CON, pain intensity increased during the dynamic contractions at both sessions (Friedman test: *P* < 0.001). ^#^ *P* < 0.001 compared to 140 min (Dunn’s post-hoc test). *VAS* visual analogue scale

In both sessions the overall pain intensity and the pain intensity in the most painful leg were, as expected, higher in FMS than in CON throughout the microdialysis (data during the trauma phase not shown). There were no significant changes in pain levels in the most painful leg during the first 120 min of the trauma phase in FMS at any session. In CON, the pain changed significantly during the trauma phase at the second session, i.e., after the resistance exercise (*P* = 0.011), but the post-hoc test (Dunn’s test) could not detect any significant difference between time points. During the 20 min of dynamic contractions the pain intensity increased in FMS in both sessions. The post-hoc test showed significantly higher pain at 155 min (data not shown) and 160 min compared to 140 min at both sessions (Fig. 2). There were no significant changes in CON during the dynamic contractions.

The overall pain_AV_ during dynamic contractions had decreased in FMS after the 15 weeks of resistance exercise, but not the pain_AV_ in the exercising leg. There were no significant differences in overall pain_AV_ or in the exercising leg in CON between sessions (Table 2).

**Table 2:** Overall pain intensity, pain intensity in exercising leg, and fatigue in 24 patients with fibromyalgia (FMS) and 27 healthy controls (CON) before and after 15 weeks of resistance exercise twice per week, as well as the *P* value for within-group comparison (Wilcoxon test)

		FMS		*P* value	CON		*P* value
		Before	After		Before	After	
Pain	Overall (0–100)	53.5 (29.3)	50.0 (23.5)	***0.025***	0 (0)	0 (0)	0.723
	Leg (0–100)	52.8 (24.4)	42.8 (32.7)	0.153	0 (1.5)	0 (0)	0.751
Fatigue	Borg RPE	13.3 (2.7)	11.5 (4.0)	0.095	7.8 (2.8)	7.4 (3.0)	0.689

The figures represent the average of data sampled during microdialysis (140-220 min), which included 20 min of dynamic exercise. Data are presented as median (interquartile range)

All variables differed significantly between groups at both sessions (*P* < 0.001; Mann-Whitney *U* test)

Bold italic figures denote significant differences

The level of fatigue induced by the dynamic contractions increased in both groups and in both sessions (Friedman test: *P* < 0.001). The post-hoc test showed that it was higher than at baseline at all time points in FMS before exercise (*P* < 0.003) and at 150 min, 155 min, and 160 min after exercise (*P* < 0.001). In CON it was higher than baseline at 150 min, 155 min, and 160 min before exercise (*P* < 0.018) and at 155 min and 160 min after exercise (*P* < 0.035).

The level of fatigue_AV_ was higher in FMS compared to CON at both sessions (*P* < 0.001). There were no significant differences in fatigue_AV_ between sessions in any group (Table 2).

### Correlations between dialysate cytokines and pain variables

In FMS there were moderately strong correlations between the baseline dialysate level of IL-6 and overall pain intensity at baseline (*r*_*s*_ = 0.48, *P* < 0.05, *n* = 24). There were no significant correlations between baseline pain and cytokine levels in CON. Neither were there any correlations between cytokine_AV_ levels and pain_AV_/fatigue_AV_ levels in response to dynamic contractions, or between changes of cytokine and pain/fatigue levels immediately post-contractions in any group.

### Muscle force and endurance

Table 3 shows muscle force before and after resistance exercise. Before exercise the FMS group performed less well on the 6MWT than CON. They also had less force in the elbow flexors than CON, but there were no differences in knee extension force in the right and left legs between groups. However, the knee extension force of the most painful leg was lower in FMS than CON.

**Table 3:** Static strength of the arm flexor and knee extensor muscles, and distance walked during a 6-min walk test (6MWT) in patients with fibromyalgia (FMS) and healthy controls (CON) before and after 15 weeks of resistance exercise twice per week, as well as the *P* value for within-group comparison (Wilcoxon test)

		FMS		*P* value	CON		*P* value
		Before	After		Before	After	
Elbow flexion force (kg)							
	Right	11.9 (6.5)^#^	14.3 (6.7)*	***<0.001***	18.4 (6.2)	16.9 (6.3)	0.845
	Left	11.6 (7.3)^#^	14.2 (7.4)*	***0.007***	16.4 (5.9)	17.3 (4.3)	0.845
Knee extension force (N)							
	Right	322 (117)	340 (122)	0.153	380 (134)	406 (101)	0.248
	Left	310 (108)	328 (138)	0.066	348 (125)	365 (106)	0.248
	Most painful	307 (133)*	338 (110)*	0.307	380 (134)	405 (87)	0.248
6MWT (m)		561 (66) ^†^	570 (100)^#^	0.153	633 (100)	665 (62)	0.054

Data are presented as median (interquartile range)

Bold italic figures denote significant differences

Between group comparisons: ^#^ *P* < 0.001, ^†^ *P* < 0.01 and *P < 0.05 (Mann-Whitney *U* test)

Both groups had a high participation rate in the resistance exercise. In the FMS group the median (IQR) participation rate was 95 % (111 %) and in CON 97 % (7 %). Although both groups improved their muscle force and endurance, intra-group comparison showed that only the elbow flexion test in FMS reached statistical significance (Table 3). As before the intervention, between-groups comparison showed significant differences in the 6MWT, arm flexion force, and knee extension force of the most painful leg, with lower values in FMS after the exercise.

### Background data

Background characteristics of the participants are presented in Table 4. There were no significant differences in anthropometric data between groups. As could be expected, the overall pain intensity was higher and average PPT for all sites combined were lower in FMS compared to CON. Patients also had higher HAD depression and HAD anxiety scores than CON, although they were in the normal range (<10) in both groups. FMS further scored lower on the SF-36-PSC and SF-36-MSC than CON.

**Table 4:** Background data in 24 patients with fibromyalgia (FMS) and 27 healthy controls (CON) obtained from questionnaires and clinical examination, and significance level (*P* value) for group comparison (Mann-Whitney *U* test)

Variables	FMS	CON	*P* value
Age (years	57.0 (9.0)	57.0 (9.0)	0.800
Height (m)	1.65 (9.8)	1.65 (6.0)	0.743
Weight (kg)	68 (15)	63 (13)	0.091
BMI (kg/m^2^)	25.4 (6.6)	23.5 (4.5)	0.053
BP diastolic (mm Hg)	85 (10)	82.1 (8.3)	0.555
BP systolic (mm Hg)	133 (24)	131 (16)	0.494
Pain duration (years)	15.2 (8.2)	NA	
VAS overall (0–100)	52 (39)	0 (2)	***<0.001***
Tender points (*n*)	15.9 (1.4)	NA	
PPT-all sites (kPa)	180 (112)	326 (100)	***<0.001***
HAD-Depression (0–21)	6.0 (5.6)	1.6 (1.8)	***<0.001***
HAD-Anxiety (0–21)	6.0 (7.0)	3.4 (3.1)	***0.012***
SF36-PSC (0–100)	30.0 (9.3)	54.4 (4.5)	***<0.001***
SF36-MSC (0–100)	44.7 (18.5)	51.2 (6.0)	***0.019***

Data are presented as median (interquartile range)

Bold italic figures denote significant differences

*BMI* body mass index, *BP* blood pressure, *HADS-Anxiety* Hospital Anxiety and Depression Scale—anxiety subscale, *HADS-Depression* Hospital Anxiety and Depression Scale—depression subscale, *NA* not applicable, *PPT-all sites* pressure pain thresholds (mean for all sites), *SF36-PCS* Short-Form Health Survey 36-physical summary component, *SF36-MSC* Short-Form Health Survey 36-mental (psychological) summary component, *VAS* visual analogue scale

## Discussion

This is the first study that has explored both the interstitial muscle levels and systemic levels of cytokines in FMS before and after a resistance exercise. Although overall pain intensity had decreased and arm muscle force improved after exercise, there were no significant changes of pain intensity or muscle force in the exercising leg. Neither were there any significant changes in muscle or plasma cytokine levels. Thus, 15 weeks of resistance exercise twice per week did not influence the peripheral release of cytokines.

Several studies have shown that exercise may have an anti-inflammatory effect on various inflammatory conditions, for example osteoarthritis of the knee [47, 48], by reducing systemic cytokine levels [49]. Also, in FMS, some previous studies, mainly from the same group, indicate that an exercise intervention may reduce systemic cytokine levels. Ortega and co-workers in a pilot study of FMS patients reported that 4 months of aquatic exercise three times per week reduced elevated serum IL-8 levels [50]. They later followed up on the study by showing that after 8 months of aquatic exercise twice per week there were no differences in the spontaneous and lipopolysaccharide (LPS)-stimulated monocyte release of IL-1β or IL-6 in FMS compared to healthy controls, but a lower release of TNF and higher release of IL-10. At baseline, before exercise, the release of all these cytokines was higher in FMS [51]. Another study from the group reported significantly reduced monocyte release of IL-8 and noradrenaline in FMS after 8 months of aquatic exercise compared to FMS patients that had not been exercising [52]. Combined, these results indicate that an exercise intervention may reduce systemic IL-8, but increase IL-10. Available evidence from a systematic review (mainly based on the studies above) suggests that exercise interventions may reduce systemic IL-6 and IL-8 levels [30]. Thus, these results differ from the results of the present study regarding muscle levels of pro-inflammatory cytokines. The results cannot be directly compared as the previous studies investigated circulating cytokine levels. However, plasma cytokine levels did not change after the resistance exercise either. The reason for this difference could be due to methodological issues; for example, aquatic exercise compared to resistance exercise, duration of exercise, and so forth. The former studies also included a limited number of patients (*n* < 14), which is why the power was probably low which questions the validity of the results. Also, the present study had a limited patient sample (*n* = 24), although most other microdialysis studies studying muscle release of biomarkers in chronic pain conditions have had similar sample sizes [17, 19–21]. However, it would be feasible and warranted to conduct larger studies on the effect of exercise on plasma levels of cytokines. In addition, even if the results from the main study of this randomized controlled trial showed a positive effect on FMS symptoms [33], the pain and fatigue as well as muscle force in the exercising leg were unaffected by the resistance exercise. Thus, the exercise might also have been of too low intensity to alter muscle cytokine levels. Bjersing and co-workers measured serum and cerebrospinal fluid levels of biomarkers, among them IL-6 and IL-8, before and after 15 weeks of Nordic walking in 49 FMS patients [31] and found no significant changes, in line with the results of the present study. They further did not find any effects of the exercise on 6MWT or pain intensity.

In concordance with previous microdialysis studies [29, 53] and biopsy studies [54, 55], the interstitial levels of IL-6 and IL-8 increased in response to dynamic contractions. Baseline levels of IL-6 and IL-8 were higher in dialysate than in plasma, and the levels did not correlate. This shows that IL-6 and IL-8 are released locally in the muscle tissue. Indeed, both IL-6 and IL-8 are regarded as myokines, i.e., cytokines that are released from myocytes. Also, TNF increased in response to dynamic contractions but only in CON. TNF is normally not regarded as a myokine as it does not increase after exercise, if not highly strenuous or prolonged [56]. The dialysate levels of TNF were lower than plasma levels, which refutes that local release would have any effect on plasma levels. Even if we cannot draw any conclusions about plasma levels of cytokines in response to dynamic contractions in this study, as they were only assessed before microdialysis, it is well known that plasma IL-6 increases after exercise [27]. This is not related to muscle damage, as shown also by low-intensity concentric contractions leading to increased plasma IL-6 levels [27]. In FMS, plasma and serum cytokine levels of IL-8 were reported to be decreased after 45 min of moderate cycling [29] and after an exhaustive treadmill exercise lasting approximately 20 min [6]. The latter study also reported increased serum IL-6 and TNF levels and a blunted response of IL-10. The increase in IL-6 is in concordance with the dialysate data in this study, but the results regarding IL-8 differ. Other studies reported increased gene expression of IL-10, but not IL-6 or TNF, up to 48 h after a 25-min cycling exercise in FMS comorbid with chronic fatigue syndrome [57], but no change in patients with FMS only [58]. Hence, even if studies are not directly comparable because of different methodologies, the diverging results across studies make conclusions about the release of pro-inflammatory cytokines due to dynamic contractions in FMS impossible at present.

The positive correlations between overall pain intensity and muscle IL-6 may seemingly indicate a role for cytokines in the local muscle pathophysiology of FMS. On the contrary, the increased dialysate levels of IL-6 and IL-8 immediately after dynamic contractions did not correlate with the increase in muscle pain or fatigue levels at any visit, which speaks against this. Thus, mechanisms other than muscle release of cytokines probably account for the increase in pain and fatigue during contractions. However, circulating cytokines, especially IL-8, released mainly from other sources may still be involved in FMS pathology as has been reported in many previous studies [59].

Few previous studies have explored muscle cytokine levels with microdialysis. Of the pro-inflammatory cytokines analyzed in this study, IL-1β in dialysate was detectable in less than 50 % of samples and in very low levels both in FMS and CON, whereas the other cytokines were detectable in the majority of samples. This shows that it is possible to recover cytokines from muscle tissue with microdialysis even without adding dextran or albumin, albeit this might have improved recovery [60, 61].

Insertion of the microdialysis catheter usually disrupts the balance of substances in the tissue, which takes time to normalize. For smaller molecules, such as serotonin, glutamate and metabolites, 2 h of stabilization has been shown to be sufficient in intramuscular microdialysis [17]. For cytokines the pattern may be different. One study reported about 30 % increases in IL-6 and IL-8 levels 6 h after insertion of a microdialysis catheter in the vastus lateralis muscle of healthy volunteers [62]. Also, for IL-6 and IL-8 in this study there was a general tendency to an increase in muscle levels with time. This could, of course, also be an effect of the dynamic contractions, as an increased release of both these cytokines was found in the dialysate sample obtained directly after the contractions. However, this was a transient increase and in the following sample the levels did not differ from baseline. Nevertheless, it is not known if a steady state is reached even after 6 h so it might not be feasible in a clinical situation to prolong the microdialysis to reach a steady state.

The strength of the present study is that the local release of cytokines was measured (with microdialysis) in a muscle (vastus lateralis) that had been subjected to resistance exercise twice per week for 15 weeks. The microdialysis method also allows continuous measurements in response to, for example, muscle contractions, as in the present study. No previous studies have made similar attempts, even if biopsy studies before and after acute exercise have been performed [54, 55]. Another strength is that the study population represents FMS patients in the general population and not patients from tertiary care clinics. This may explain why the depression levels were within the normal range, contrary to other studies in FMS [63]. Among the study limitations is the sample size. Even if it was larger than previous studies regarding exercise effects on cytokine levels in FMS [50–52], the sample was still somewhat limited. Furthermore, to be able to compare cytokine levels before and after the intervention, the average of the dialysate levels during the dynamic contractions was used. This might have disguised subtle changes between sessions. However, as shown in Fig. 1, the levels of the cytokines showed a similar pattern at both sessions which why this is less likely. No attempts were made to match the groups for variables other than age. One can therefore argue that any changes in outcome measures could be due to improvement in physical activity in FMS that was without significance for the pathophysiology. However, participants in both groups were physically inactive, and volunteered to participate as they wanted to start exercising. Finally, only pro-inflammatory cytokines were analyzed. Due to the recent findings of reduced IL-10 in response to exercise it would have been interesting to also analyze IL-10 and other anti-inflammatory cytokines such as IL-4 and IL-13.

## Conclusion

Although 15 weeks of progressive resistance exercise improved arm muscle force and reduced overall pain in FMS it neither altered the muscle levels of IL-1β, IL-6, IL-8, or TNF, nor pain and fatigue levels in the vastus lateralis muscle. However, TNF was lower in FMS than CON and increased during dynamic contractions only in CON.

## Abbreviations

%CV, percent coefficient of variation; 6MWT, 6-minute walk test; ACR, American College of Rheumatology; CON, healthy controls; CSF, cerebrospinal fluid; FMS, fibromyalgia syndrome; HADS, Hospital Anxiety and Depression Scale; IGF-1, insulin growth factor-1; IL, interleukin; IQR, interquartile range; MVC, maximum voluntary capacity; NSAID, non-steroidal anti-inflammatory drug; PPT, pressure pain threshold; SF36, Short-Form Health Survey (PSC: physical summary component, MSC: mental summary component); SSRI, selective serotonin reuptake inhibitors; TNF, tumor necrosis factor; VAS, visual analogue scale
